# Socioeconomic Inequalities of Undiagnosed Diabetes in a Resource-Poor Setting: Insights from the Cross-Sectional Bangladesh Demographic and Health Survey 2011

**DOI:** 10.3390/ijerph16010115

**Published:** 2019-01-03

**Authors:** Md. Mehedi Hasan, Fariha Tasnim, Md. Tariqujjaman, Sayem Ahmed

**Affiliations:** 1 Institute for Social Science Research, The University of Queensland, Indooroopilly 4068, Queensland, Australia; 2 Maternal and Child Health Division, International Centre for Diarrhoeal Disease Research, Bangladesh (icddr,b), 1212 Dhaka, Bangladesh; fariharuhi@gmail.com; 3 Nutrition and Clinical Services Division, International Centre for Diarrhoeal Disease Research, Bangladesh (icddr,b), 1212 Dhaka, Bangladesh; tariq.sbidu@gmail.com; 4 Health Systems and Population Studies Division, International Centre for Diarrhoeal Disease Research, Bangladesh (icddr,b), 1212 Dhaka, Bangladesh; sayemahmed@icddrb.org; 5 Health Economics and Policy Research Group, Department of Learning, Informatics, Management and Ethics, Karolinska Institute, SE-171 77 Stockholm, Sweden

**Keywords:** undiagnosed diabetes, socioeconomic condition, inequality, odds ratio, concentration index, Bangladesh

## Abstract

Diabetes mellitus is rising disproportionately but is not frequently diagnosed until complications appear, which results in adverse health consequences. We estimated the prevalence of undiagnosed diabetes among adult diabetic patients and associated socioeconomic inequalities in Bangladesh. We used nationally representative cross-sectional Bangladesh Demographic and Health Survey (BDHS) 2011 data. Among patients with diabetes, we identified undiagnosed cases as having fasting plasma glucose ≥ 7.0 mmol/L, never having taken prescribed medicine and being told by health professionals. Among 938 patients with diabetes, 53.4% remained undiagnosed. The poorest (75.9%) and rural (59.0%) patients had significantly higher undiagnosed cases than the richest (36.0%) and urban (42.5%), respectively. Multiple logistic regression analysis revealed that the likelihood of being undiagnosed was lower among patients with age ≥ 70 years vs. 35–39 years (adjusted odds ratio (AOR) = 0.35; 95% confidence interval (CI) 0.19, 0.64) and patients with higher education vs. no education (AOR = 0.36; 95% CI 0.21, 0.62). Conversely, a high level of physical activity and being in a poor socioeconomic quintile were associated with a higher risk of remaining undiagnosed for diabetes. The Concentration Index (C) also showed that undiagnosed diabetes was largely distributed among the socioeconomically worse-off group in Bangladesh (C = −0.35). Nationwide diabetes screening programs may reduce this problem in Bangladesh and other similar low-income settings.

## 1. Introduction

The disproportionate increase in the prevalence of non-communicable diseases (NCDs) has become a major public health shortcoming globally, especially in developing countries [1]. Among NCDs, diabetes mellitus is considered one of the major global public health challenges of the twenty-first century [2]. Globally, 285 million adults were affected by diabetes mellitus in 2010 and it is estimated that 439 million adults will be affected by 2030 [3]. In the South-East Asian region, more than 72 million adults are living with diabetes and it is predicted that the number will exceed 123 million by 2035 [4]. In Bangladesh, the prevalence of diabetes is 7.4%, higher among males compared to females, and is increasing over time [5].

The global NCD epidemic, especially diabetes mellitus, is increasing rapidly due to several life-style related factors including unhealthy food habits, inadequate physical activity, high body mass index (BMI), and abuse of substances operated through high blood pressure, elevated blood glucose and plasma lipid levels [6]. The shift in population age structure—an increasing proportion of the older aged population and rapid urbanization—stimulates the NCD risk [7].

Life-style related behaviors, healthcare-related knowledge and accessibility are influenced by socioeconomic status (SES) and this likely drives a lack in acquiring diabetes-related awareness and a willingness to diagnose diabetes, which is important in order to control this disease among divergent people [8,9]. Epidemiological studies have repeatedly confirmed the inverse association between the prevalence of diabetes and SES [10,11,12,13].

Diabetes has multifaceted health consequences. In addition, patients may experience sudden unfortunate health hazards if it remains undiagnosed. Diabetes may lead to a serious problem in the later life of patients if it was undiagnosed for a long time. These patients are at greater risk of experiencing a stroke, coronary heart disease, dyslipidemia and peripheral vascular disease [14]. Previous studies have estimated the prevalence and identified potential risk factors of diabetes in Bangladesh [15,16]. However, limited studies have reported the prevalence of undiagnosed diabetes and associated socioeconomic inequalities in Bangladesh. Therefore, we aimed to estimate the prevalence of undiagnosed diabetes among adult diabetic patients and related socioeconomic inequalities in Bangladesh.

## 2. Materials and Methods

### 2.1. Participants and Procedures

**Figure 1:**
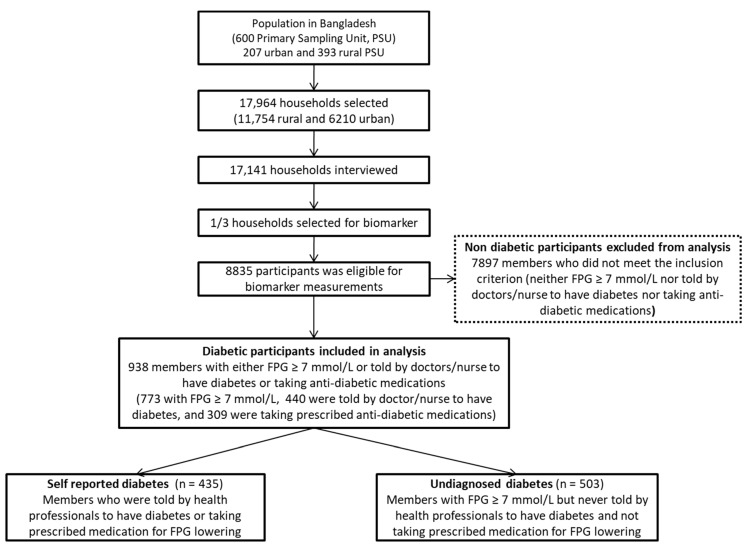
Selection of participants for this study. FPG = fasting plasma glucose.

We used nationally representative cross-sectional Bangladesh Demographic and Health Survey (BDHS) 2011 data to conduct this study. Among the seven DHSs conducted in Bangladesh so far, the 2011 BDHS is the only survey that collected data on fasting plasma glucose (FPG) for measuring diabetes at the national level. In 2011, the BDHS employed a two-stage stratified sampling procedure to collect the data. In the first stage, the BDHS selected 600 (207 in urban and 393 in rural) enumeration areas (EAs), with probability proportional to the EA size. On average, 30 households were selected systematically from each of the EAs when sampling was done at the second stage. In this cross-sectional survey, a total of 17,141 households comprising 83,731 household members were included. Among them, a total of 8835 participants of both sexes aged 35+ years were eligible for biomarker measurements. Among them, we identified three non-mutually exclusive groups: 773 cases with FPG ≥ 7.0 mmol/L, 440 cases who were told by health professionals that they have diabetes, and 309 cases who were taking prescribed anti-diabetic medicines. We finally included 938 cases whose FPG ≥ 7.0 mmol/L or who were ever told by health professionals (doctor or nurse) that they have diabetes, or who were taking anti-diabetic medicines. Hence, 7897 cases without the above conditions were dropped to confirm that we were dealing with patients with diabetes by FPG measurement and medical assessment (medical history or medicine intake). Sample selection for the study is detailed in Figure 1.

### 2.2. Outcome Variable

In the BDHS 2011, the FPG level of the participants was used for measuring diabetes. The BDHS used HemoCue 201+ blood glucose analyzers to collect a blood sample in a capillary, and this was obtained from the swab-cleaned middle or ring finger of the respondent if s/he was fasting during the interview and if not, an appointment was made to collect a blood sample while fasting. The World Health Organization (WHO) recommended cut-offs were used to define diabetes based on the FPG level of participants and was considered as normal (not diabetic) if s/he had an FPG level within 3.9–6.0 mmol/L, pre-diabetic if FPG was within 6.1–6.9 mmol/L, and diabetic if FPG was 7.0 mmol/L or more [17]. However, this study only considered patients with diabetes (participants whose FPG was 7.0 mmol/L or more, those who took prescribed medicine or those who were told by health professionals (doctor/nurse) that they had diabetes). The primary outcome variable of our study was undiagnosed diabetes. Among the patients with diabetes, the patients with undiagnosed diabetes were identified if his/her FPG was ≥ 7.0 mmol/L, they had never taken prescribed medicine and they had never been told that they had diabetes by a health professional [18].

### 2.3. Explanatory Variables

The socioeconomic status of households was measured by wealth quintiles and was considered as the main exposure variable of interest in this study. Based on the ownership of durable assets in the households, the socioeconomic status of the households was defined by creating asset scores estimated by using principal component analysis (PCA) and households were classified into five equal quintiles. PCA is a commonly used technique for creating scores for indices by using both continuous and categorical variables. Although the PCA technique is traditionally applied for continuous variables, it is valid for the parallel use of continuous and binary data such as ownership of assets [19]. Higher asset scores of the asset index indicate more affluent households. We included patient age (categorized as: 35–39 years, 40–44 years, 45–49 years, 50–54 years, 55–59 years, 60–64 years, 65–69 years and ≥70 years), sex (male and female), educational status (categorized as: no education, primary, secondary and higher), marital status (categorized as: currently married and divorced/widowed/separated), nutritional status (categorized as: thin (BMI < 18.5 kg/m^2^), normal (BMI ≥ 18.5 kg/m^2^ but BMI < 25 kg/m^2^) and overweight/obese (BMI ≥ 25 kg/m^2^)), physical activity levels at work (categorized as: “work that requires heavy physical activity”, “work that requires moderate physical activity” and “work that requires light physical activity”), households having children (dichotomous: 0 = “no child” and 1 = “has child”), administrative divisions (e.g., Barisal, Chittagong, Dhaka, Khulna, Rajshahi, Rangpur and Sylhet), and place of residence (e.g., urban and rural) as confounding factors.

### 2.4. Statistical Analyses

Bivariate analysis using a Chi-square test was employed to investigate the association between the prevalence of undiagnosed diabetes among adult diabetic patients and background characteristics. This prevalence estimation was done taking complex survey design into account for capturing variations due to the weighting and design of the BDHS 2011.

Multiple binary logistic regression analysis was carried out to explore the potential determinants of undiagnosed diabetes. In a multiple regression model, we entered variables that were significantly associated (*p*-value < 0.05) with undiagnosed diabetes in simple logistic regression models as independent variables. The results of simple and multiple logistic regression analysis were presented respectively in terms of odds ratios (OR) and adjusted odds ratios (AOR) along with their respective 95% confidence intervals (CI). Variations in the errors due to clustering were also controlled when performing regression analyses by using the “cluster” command. We also checked the variation inflation factors (vif) to detect multicollinearity among the covariates in the multiple regression model. We found that vif < 2.20 for all the variables that we entered in the model, which represents the absence of multicollinearity among the covariates [20].

We estimated the concentration index (C) to measure the magnitude of inequalities in the prevalence of undiagnosed diabetes by asset-based socioeconomic status. The C is defined as twice the area between the concentration curve and the line of equality. The value of the C usually ranges from −1 to +1; a positive value implies the prevalence is more concentrated among better-off individuals, and a negative value implies the prevalence is more concentrated among the less affluent population [21,22]. Since our outcome variable is a bounded variable (e.g., the prevalence of undiagnosed diabetes among adult diabetic patients ranges between 0 and 1), the value of the standard C may not always be within −1 to +1 [23]. To keep intact of the invariance property of relative C, Wagstaff proposed a modified C for binary outcome variables by rescaling the standard C [24]. We measured the inequality by calculating the modified C of Wagstaff by using the “conindex” command in STATA [25]. We also estimated the corrected version of C proposed by Erreygers in 2009 [26]. STATA (version 13) was used to perform all the analyses.

### 2.5. Ethical Clearance

The BDHS survey methodology and questionnaire was reviewed and approved by the ICF Institutional Review Board. BDHS obtained written consent from the respondents before conducting the survey. Therefore, separate ethical approval was not required for this study.

## 3. Results

### 3.1. General Characteristics of the Study Participants

Among the patients studied, the average age was 52.7 years (95% CI 51.8, 53.6). There were almost equal numbers of males (49.0%; 95% CI 45.7%, 52.4%) and females (51.0%; 95% CI 47.6%, 54.3%) in the sample (Table 1). However, more than one-third of the patients had no education (35.5%; 95% CI 31.4%, 39.5%). One in every four patients was overweight/obese (BMI ≥ 25 kg/m^2^). Findings showed that 63% (95% CI 59.7%, 66.3%) of patients were involved in work that required light physical activity. A higher number of patients resided in a rural area than an urban area. The highest number of patients belonged to richest wealth quintile (39.1%; 95% CI 35.0%, 43.2%) and the lowest number of patients belonged to the poorest wealth quintile (12.1%; 95% CI 9.4%, 14.9%).

### 3.2. Prevalence of Undiagnosed Diabetes among Adult Diabetic Patients

**Figure 2:**
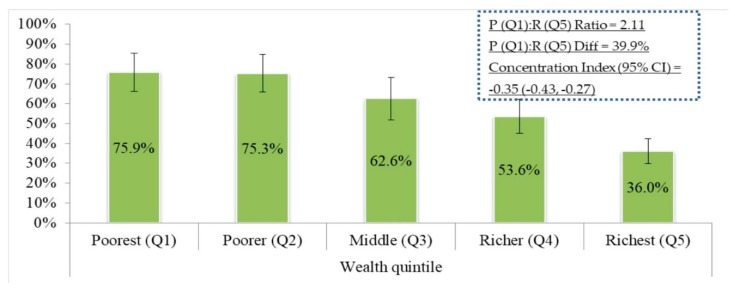
Socioeconomic inequalities of undiagnosed diabetes in Bangladesh.

According to our study, 938 cases were diagnosed as having diabetes with a mean FPG of 0.806 mmol/L (standard error 0.01). Among these patients, we found that 503 (53.4%) patients were undiagnosed during the survey (Table 2). The prevalence of undiagnosed diabetes among adult diabetic patients varied across the patients’ education and BMI classification (*p*-value < 0.001). A greater prevalence of undiagnosed diabetes was observed among patients with no education (67.2%) compared to patients with higher education (33.2%) and patients who were thin (66.8%) compared to patients with normal BMI (55.1%). Compared to patients of the richest wealth quintile (36.0%), the prevalence of undiagnosed diabetes was higher among patients of the poorest (75.9%) and poorer (75.3%) quintiles (Figure 2). The rate of undiagnosed diabetes was higher among patients who were involved in heavy physical activity (71.3%) compared to those whose work requires light physical activity (50.2%). Notable variations in the prevalence of undiagnosed diabetes were also observed across administrative divisions (*p*-value < 0.001) (Table 2).

### 3.3. Determinants of Undiagnosed Diabetes

We found that patient age, education, BMI, physical activity, administrative division, place of residence and wealth quintiles were significantly associated with the prevalence of undiagnosed diabetes among adult diabetic patients in unadjusted regression analysis.

The good fitted (log pseudo-likelihood = −547.21912; pseudo R-square = 0.1203; Wald chi-square = 108.17; *p*-value <0.001) multiple binary logistic regression model showed that the age of patients was associated with undiagnosed diabetes. Elderly patients had a lower likelihood of being undiagnosed than patients with age 35–39 years (for patients with age ≥ 70 years: AOR = 0.35; 95% CI 0.19, 0.64). Patients who received primary education (AOR = 0.63; 95% CI 0.43, 0.93), secondary education (AOR = 0.48; 95% CI 0.30, 0.76) and higher education (AOR = 0.36; 95% CI 0.21, 0.62) were less likely to have undiagnosed diabetes compared to patients with no education.

Patients whose work required moderate physical activity (AOR = 1.53; 95% CI 1.01, 2.32), as well as heavy physical activity (AOR = 1.73; 95% CI 1.14, 2.64), were more likely to have undiagnosed diabetes than those involved in light physical activity. Moreover, patients with a poorer socioeconomic status had a high chance of having undiagnosed diabetes compared to higher socioeconomic quintiles. Patients of the poorest and poorer wealth quintiles were 4.08 (AOR = 4.08; 95% CI 2.12, 7.86) and 3.52 (AOR = 3.52; 95% CI 1.89, 6.54) times more likely to have undiagnosed diabetes than patients of the richest wealth quintiles.

Moreover, significant geographic variation was evident in the prevalence of undiagnosed diabetes among adult diabetic patients. Residents of Dhaka (AOR = 0.36; 95% CI 0.21, 0.64), Khulna (AOR = 0.51; 95% CI 0.28, 0.92) and Rajshahi (AOR = 0.38; 95% CI 0.22, 0.67) had a 64%, 49% and 62% less chance of having undiagnosed diabetes, respectively, than patients from Barisal (Table 3).

### 3.4. Socioeconomic Inequalities in Undiagnosed Diabetes

We found that the prevalence of undiagnosed diabetes among adult diabetic patients was disproportionately distributed among worse-off socioeconomic groups (C = −0.35; 95% CI −0.43, −0.27). The absolute difference in the distribution of undiagnosed diabetes was 39.9% between poorest and richest. Moreover, we found a 2.11 poor (quintile 1): rich (quintile 5) ratio for the distribution in the prevalence of undiagnosed diabetes in Bangladesh (Figure 2). The C estimate (C = −0.35) using Erreyger’s corrected approach showed similar results.

## 4. Discussion

Undiagnosed diabetes may lead to adverse health consequences. This study adopted nationally representative survey data (BDHS 2011) to estimate the prevalence of undiagnosed diabetes among adult diabetic patients, along with the risk factors associated with it.

The BDHS 2011 estimated that the diabetes prevalence among adults is 11.2% in Bangladesh, of which more than half of the study participants were identified as having a FPG ≥ 7.0 mmol/L who were not screened or diagnosed with diabetes before the survey. This might be due to the lack of accessibility, availability and utilization of healthcare services. It is evident from the Bangladesh Health Facility Survey 2014 that services for diabetes are offered from district to union level but diabetes diagnosis capacity are limited to Upazila Health Complex (sub-district level). Only one-third of the district and Upazila-level health facilities had diagnostic materials [27]. This indicates that in a resource-poor setting like Bangladesh, less coverage of healthcare services and insufficient screening materials may lead to a higher proportion of undiagnosed diabetes. Nonetheless, knowledge, perception and the management of diabetes are influenced by people’s access to healthcare centers [28]. Therefore, patients living away from healthcare centers that provide diabetes care may have a poorer understanding of the importance of diabetes screening and its management. Moreover, people do not usually seek care unless they are exposed to severe health hazards and therefore early symptoms of diabetes are often ignored [29].

Few studies have been conducted on undiagnosed diabetes in low- and middle-income countries to date. Latif et al. reported in 2011 that among patients with diabetes, nearly half were undiagnosed in Bangladesh [30]. A similar prevalence of undiagnosed diabetes has been observed in a study conducted in the rural residences of Bangladesh [31]. Our results align with these findings. Around one-third of diabetes cases are undiagnosed worldwide [14], which is lower than the current rate of undiagnosed diabetes among Bangladeshis as estimated in this study. However, findings from several studies suggest that this ratio is lower in developed countries [17,32,33,34,35,36]. In a study conducted in Quebec, Canada, 40% of diabetes patients were found to be undiagnosed [32]. In the USA, more than one-third of patients were found to be undiagnosed diabetics in two different studies conducted on adolescents and the adult Mexican border populations, respectively [33,34]. Seven million undiagnosed diabetic patients were identified by 2010 [35]. In contrast, less than 10% undiagnosed diabetes was found in Norway and England [18,36].

A number of previous studies have reported a higher risk of diagnosed diabetes among people: in older age, residing in an urban area, with a higher education level, who are overweight, and who were involved in less physical activity [16,18,37,38]. We found that people aged less than 50 years, rural dwellers, less educated, thin patients, and those who were involved in heavy physical activity were at higher risk of having undiagnosed diabetes. This may be because of the misconception that people who are relatively young, underweight or those involved in heavy physical activity are less likely to suffer from any severe disease. These groups may therefore be less conscious about their health status [39]. People with no educational background and living in a rural area are not aware of the symptoms of diabetes and they may not consider this as a threat to their health [40]. Therefore, even if they are exposed to one or more symptoms of diabetes, they still may not consult a healthcare provider.

Our study demonstrates that inequalities exist in the prevalence of undiagnosed diabetes across the wealth quintiles and this was more prevalent among the poor compared to the rich. This is contrary to the study conducted on patients with diabetes which found inequality in the opposite dimension [41]. Greater awareness and more utilization of healthcare benefits among the rich may be the reason behind this disparity [42]. To reduce this gap, public health strategies should concentrate more on the cost-effective allocation of resources which has to be equitable for all.

In Bangladesh, despite the risk factors of [15,16] and the inequalities in [41], diabetes prevalence was well detected at a national level, which has guided people and policy makers to control the disease. However, controlling this disease will not be meaningful unless all patients are accurately diagnosed and detected. Also, the identification of unequal distribution of patients with undiagnosed diabetes across different socioeconomic groups is essential for setting priorities and allocation of resources. The findings of this study will further guide policy makers in this aspect by taking the disparities in the distribution of undiagnosed diabetes into consideration.

We endeavoured to identify the potential risk factors by taking cluster variation into account. However, there could be residual or unmeasured confounders. We used cross-sectional data which prevents us from detecting causal relationships between undiagnosed diabetes and confounders.

To date, no large-scale study had been conducted on undiagnosed diabetes in Bangladesh. This study provides a rare opportunity to estimate the prevalence of undiagnosed diabetes as a major threat to health outcomes in Bangladesh through a nationally representative survey. Furthermore, risk factors were identified using the odds ratio, which is widely accepted. The incorporation of a biomarker test in the BDHS 2011 provided evidence of glucose abnormalities in a substantial proportion of individuals, indicating that screening practices for diabetes need to be widened with the greatest possible coverage of the population.

## 5. Conclusions

Undiagnosed diabetes is highly prevalent among adult diabetic patients in Bangladesh. The findings of this study suggest initiatives need to be taken for diabetes screening, especially among the marginalized society. To ensure routine screening, a surveillance system with feasible biomarker testing can help in tracking disease incidence and the people remaining undiagnosed. Policies and programs should concentrate on capturing the highest domain of underprivileged population under surveillance to ensure routine screening at the lowest possible costs. This may further reduce the disparities in the diagnosis of diabetes among the different socioeconomic groups. Healthy lifestyle practice is another precautionary measure to deal with this undiagnosed problem. Efforts to improve screening are crucial to target adults who are poor, have a lower education level and those involved in heavy physical activity, given their higher risk of being undiagnosed. Findings from this study may contribute to preparing or reformulating policy for reducing the prevalence of undiagnosed diabetes in Bangladesh, and other low- and middle-income countries with poor healthcare infrastructure.

## Figures and Tables

**Table 1:** Characteristics of the study participants.

Variables	Frequency (n = 938)	Percentage (%)	95% Confidence Interval	
			Lower Bound (%)	Upper Bound (%)
**Age in years**				
35–39	136	14.6	11.9	17.3
40–44	147	15.6	13.0	18.2
45–49	151	15.7	13.0	18.4
50–54	120	12.8	10.2	15.3
55–59	123	13.3	10.9	15.8
60–64	82	8.8	6.8	10.8
65–69	53	6.4	4.5	8.3
≥70	126	12.9	10.3	15.4
**Sex**				
Male	468	49.0	45.7	52.4
Female	470	51.0	47.6	54.3
**Educational status**				
No education	297	35.5	31.4	39.5
Primary	260	27.5	24.1	30.9
Secondary	221	21.5	18.5	24.4
Higher	160	15.5	12.6	18.5
**Marital status**				
Currently married	777	83.4	80.6	86.2
Divorced/separated/others	161	16.6	13.8	19.4
**Nutritional status**				
Normal	495	55.1	51.8	58.5
Thin	154	18.4	15.2	21.5
Overweight/obese	261	26.5	23.3	29.7
**Physical activity levels at work**				
Light physical activity	576	63.0	59.7	66.3
Moderate physical activity	175	18.4	15.5	21.3
Heavy physical activity	178	18.6	15.7	21.5
**Households with children**				
No child	589	64.2	60.4	68.0
Has child	349	35.8	32.0	39.6
**Wealth quintil**e				
Poorest	101	12.1	9.4	14.9
Poorer	98	12.3	9.5	15.2
Middle	125	14.3	11.6	17.1
Richer	191	22.1	18.6	25.5
Richest	423	39.1	35.0	43.2
**Division**				
Barisal	110	5.8	4.9	6.8
Chittagong	171	21.5	18.4	24.5
Dhaka	168	33.7	30.1	37.4
Khulna	123	9.7	8.4	11.0
Rajshahi	132	14.1	12.1	16.1
Rangpur	103	9.1	7.5	10.8
Sylhet	131	6.0	5.0	6.9
**Place of residence**				
Urban	420	33.9	30.5	37.3
Rural	518	66.1	62.7	69.5

**Table 2:** Differentials in the prevalence of undiagnosed diabetes among adult diabetic patients across background characteristics.

Variables	Undiagnosed Diabetes (%)	95% Confidence Interval		Chi-Square Statistic (*p*-value)
		Lower Bound	Upper Bound	
**Age in years**				12.1 (0.2207)
35–39	62.3	52.8	71.9	
40–44	55.7	47.0	64.3	
45–49	52.4	42.8	62.1	
50–54	50.1	40.3	60.0	
55–59	56.3	46.4	66.2	
60–64	40.3	28.1	52.6	
65–69	47.5	32.1	63.0	
≥70	53.4	43.4	64.2	
**Sex**				4.5 (0.0496)
Male	56.9	51.5	62.2	
Female	50.0	44.8	55.2	
**Educational status**				61.0 (<0.001)
No education	67.2	60.7	73.1	
Primary	56.1	48.7	63.2	
Secondary	41.8	33.8	50.2	
Higher	33.2	25.4	42.0	
**Marital status**				0.1 (0.8388)
Currently married	53.6	49.0	58.1	
Divorced/separated/others	52.4	42.6	62.1	
**Nutritional status**				27.0 (<0.001)
Normal	55.1	49.8	60.3	
Thin	66.8	56.2	76.0	
Overweight/obese	41.2	34.2	48.7	
**Physical activity levels at work**				29.1 (<0.001)
Light physical activity	50.2	45.4	55.0	
Moderate physical activity	45.3	36.9	54.0	
Heavy physical activity	71.3	62.9	78.5	
**Households with children**				0.1 (0.7585)
No child	53.8	48.6	59.0	
Has child	52.6	46.2	58.9	
**Division**				28.4 (<0.001)
Barisal	68.0	59.2	75.8	
Chittagong	54.8	46.8	62.6	
Dhaka	44.4	35.8	53.3	
Khulna	53.1	42.8	63.1	
Rajshahi	52.9	43.8	61.8	
Rangpur	72.9	61.6	81.8	
Sylhet	56.6	44.9	67.5	
**Place of residence**				22.8 (<0.001)
Urban	42.5	35.9	49.4	
Rural	59.0	53.9	63.8	
**Overall**	**53.4**	**49.3**	**57.5**	

**Table 3:** Cluster controlled determinants of undiagnosed diabetes among adult diabetic patients in Bangladesh.

Variables	Unadjusted OR (95% CI)	Adjusted OR (95% CI)
**Age in years**		
35–39 (Reference)	1	1
40–44	0.68 (0.43, 1.09)	0.67 (0.39, 1.13)
45–49	0.64 * (0.40, 1.05)	0.53 ** (0.30, 0.90)
50–54	0.43 *** (0.26, 0.71)	0.33 *** (0.19, 0.59)
55–59	0.61 ** (0.38, 0.98)	0.57 ** (0.33, 0.99)
60–64	0.41 *** (0.24, 0.71)	0.35 *** (0.19, 0.66)
65–69	0.59 * (0.32, 1.10)	0.50 ** (0.25, 1.00)
≥70	0.55 ** (0.33, 0.91)	0.35 *** (0.19, 0.64)
**Sex**		
Male (Reference)	1	
Female	0.81 * (0.64, 1.03)	n/a
**Educational status**		
No education (Reference)	1	1
Primary	0.56 *** (0.40, 0.79)	0.63 ** (0.43, 0.93)
Secondary	0.40 *** (0.27, 0.58)	0.48 *** (0.30, 0.76)
Higher	0.27 *** (0.18, 0.41)	0.36 *** (0.21, 0.62)
**Marital status**		
Currently married (Reference)	1	
Divorced/separated/others	0.99 (0.69, 1.41)	n/a
**Nutritional status**		
Normal (Reference)	1	1
Thin	1.90 *** (1.27, 2.85)	1.17 (0.74, 1.87)
Overweight/obese	0.71 ** (0.52, 0.97)	1.04 (0.74, 1.47)
**Physical activity levels at work**		
Light physical activity (Reference)	1	1
Moderate physical activity	0.90 (0.64, 1.25)	1.53 ** (1.01, 2.32)
Heavy physical activity	2.06 *** (1.42, 3.01)	1.73 ** (1.14, 2.64)
**Households with children**		
No child (Reference)	1	
Has child	0.96 (0.73, 1.27)	n/a
**Wealth quintile**		
Richest (Reference)	1	1
Richer	2.12 *** (1.45, 3.11)	1.75 *** (1.15, 2.67)
Middle	2.65 *** (1.69, 4.15)	2.05 *** (1.21, 3.47)
Poorer	4.66 *** (2.81, 7.71)	3.52 *** (1.89, 6.54)
Poorest	6.08 *** (3.63, 10.17)	4.08 *** (2.12, 7.86)
**Division**		
Barisal (Reference)	1	1
Chittagong	0.64 * (0.40, 1.02)	0.61 * (0.36, 1.03)
Dhaka	0.41 *** (0.25, 0.69)	0.36 *** (0.21, 0.64)
Khulna	0.62 * (0.37, 1.03)	0.51 ** (0.28, 0.92)
Rajshahi	0.52 ** (0.31, 0.87)	0.38 *** (0.22, 0.67)
Rangpur	1.07 (0.58, 1.97)	0.75 (0.38, 1.51)
Sylhet	0.65 (0.38, 1.12)	0.61 (0.34, 1.11)
**Place of residence**		
Urban (Reference)	1	1
Rural	1.75 *** (1.31, 2.34)	0.89 (0.64, 1.25)

* *p*-value < 0.10, ** *p*-value < 0.05, *** *p*-value < 0.01; OR = odds ratio; CI = confidence interval.
